# Identification of Loci Controlling the Dwarfism Trait in the White Sailfin Molly (*Poecilia latipinna*) Using Genome-Wide Association Studies Based on Genotyping-By-Sequencing

**DOI:** 10.3390/genes10060418

**Published:** 2019-05-30

**Authors:** Fan Shao, Jing Liu, Mengyuan Ren, Junying Li, Haigang Bao, Changxin Wu

**Affiliations:** National Engineering Laboratory for Animal Breeding, Beijing Key Laboratory of Animal Genetic Improvement, College of Animal Science and Technology, China Agricultural University, Beijing 100193, China; autumnvan@126.com (F.S.); jing921580312@163.com (J.L.); rmy2017@163.com (M.R.); lijunying@cau.edu.cn (J.L.); chxwu@cau.edu.cn (C.W.)

**Keywords:** dwarfism, genotyping-by-sequencing, genome-wide association studies, sailfin molly, *Poecilia latipinna*, *ADAMTS like 1*, *Larp7*, *PPP3CA*

## Abstract

Dwarfism is a condition defined by low harvest weight in fish, but also results in strange body figures which may have potential for the selective breeding of new ornamental fish strains. The objectives of this study are to reveal the physiological causes of dwarfism and identify the genetic loci controlling this trait in the white sailfin molly. Skeletons of dwarf and normal sailfin mollies were observed by X-ray radioscopy and skeletal staining. Genome-wide association studies based on genotyping-by-sequencing (n = 184) were used to map candidate genomic regions associated with the dwarfism trait. Quantitative real-time PCR was performed to determine the expression level of candidate genes in normal (n = 8) and dwarf (n = 8) sailfin mollies. We found that the dwarf sailfin molly has a short and dysplastic spine in comparison to the normal fish. Two regions, located at NW_015112742.1 and NW_015113621.1, were significantly associated with the dwarfism trait. The expression level of three candidate genes, *ADAMTS like 1*, *Larp7* and *PPP3CA,* were significantly different between the dwarf and normal sailfin mollies in the hepatopancreas, with *PPP3CA* also showing significant differences in the vertebrae and *Larp7* showing significant differences in the muscle. This study identified genomic regions and candidate genes associated with the dwarfism trait in the white sailfin molly and would provide a reference to determine dwarf-causing variations.

## 1. Introduction

Dwarf ornamental fish, such as the balloon molly and dwarf swordtail, are popular in China for their lovely body shapes and graceful activities. By analyzing the skeletal morphology of the dwarf and normal swordtail fishes, Li et al. found the spine length of the dwarf swordtail to be shorter than that of the normal fish, and speculated that dwarfism in the swordtail fish was caused by a developmental defect in the spine [1]. 

Spinal deformities are a frequent phenomenon in many different fish species and can be caused by many factors during fish development, such as bacterial and parasitic infections, water environment, elevated egg incubation temperatures, pollution, nutrients, inappropriate light regimes and vaccination, etc. [2,3,4,5,6,7]. Spinal deformities often result in increased metabolic costs in the deformed fish, leading to reduced growth and low harvest weight [8]. The more deformed the spine is, the smaller the size and weight of the fish [9]. Genetic variations may play an important role in the growth and development of animal spines [10,11]. McMenamin et al. revealed a premature stop codon in the *gh1* gene of zebrafish which retarded growth [12]. Inohaya et al. reported that a DNA fragment insertion in the promoter region of the *wnt4b* gene caused fused vertebrae and shortened body length in medaka [13]. Li et al. suggested that the dwarf trait of the swordtail fish could be inherited and, in accordance with the basic characteristics of quantitative trait inheritance, might be controlled by multiple quantitative trait genes [1]. 

Genome-wide association studies (GWAS) present a popular and powerful method to map candidate loci associated with target traits [14]. High-throughput single nucleotide polymorphism (SNP) chips with thousands of SNPs uniformly distributed along the genome are usually needed to genotype SNPs and improve the effect of GWAS [15]. However, for species without economical high-density SNP chips, a simple, low-cost and efficient method, such as genotyping-by-sequencing (GBS), might be the best technology, based on second-generation sequencing technology, to obtain enough SNPs to uniformly cover the entire genome [16]. 

The sailfin molly (*Poecilia latipinna*) is a type of ornamental fish that originated from America or Mexico [17]. Generally, two types of figures are observed in the sailfin molly, one is dwarf and the other is normal. The dwarf sailfin molly, also called the balloon belly molly, is more popular than the normal sailfin molly, but the mechanism of how the balloon phenotype comes into being is still unknown. In the present study, the skeleton shape of the dwarf and normal sailfin mollies was observed and GWAS based on GBS was performed on 184 white sailfin mollies to map the genomic regions associated with the dwarfism trait. We also determined the expression levels of candidate genes within important genomic regions to screen for candidate genes which could be important in controlling the dwarfism trait.

## 2. Materials and Methods 

### 2.1. Ethics Statement 

All experimental procedures and animal use were approved by the Ethics Review Committee for Laboratory Animal Welfare and Animal Experiment of China Agricultural University (approval number: CAU20170502-3).

### 2.2. Animals and Phenotyping

**Figure 1:**
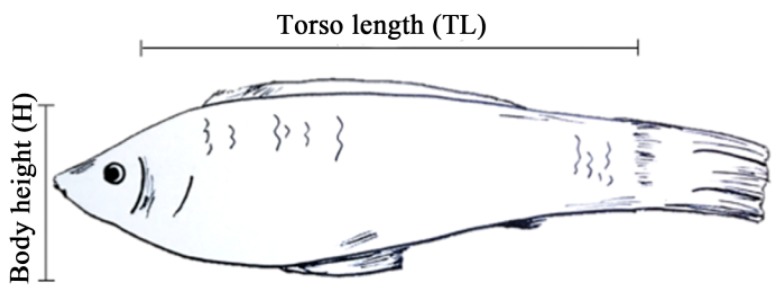
Diagram of the measured body traits.

We purchased 184 white sailfin mollies at five to six months of age from Shanghai Yuyi Tropical Ornamental Fish Farm Corporation. Classification of body shape was based on the general market classification criteria. The torso length (TL) and body height (H) of each white sailfin molly were measured using a Vernier caliper (Figure 1) and the ratio of TL and H (TL/H) was calculated and used as an index for dwarfism and for subsequent analysis. The least squares algorithm of the SAS software package (SAS 9.2, SAS Institute Inc., Cary, NC, USA) was used to analyze the phenotypic values, which were then expressed as mean ± SE.

### 2.3. Comparison of Skeletal Structures between the Dwarf Molly and the Normal Molly 

Several specimens of each body type were selected at random and their skeletal features were observed by an X-ray radioscopy inspection system (two specimens of each body type; FX Pro/FX, Carestream, USA) or skeletal staining [18] (three and five specimens of the normal and dwarf mollies, respectively).

### 2.4. Genotyping

The tail fin of each fish was collected to extract DNA. DNA extraction was performed using the TIANamp Genomic DNA Kit (cat. #DP304-02, TIANGEN, Beijing, China) according to the protocol supplied. All samples were genotyped using the genotyping-by-sequencing method [19] with some revisions. Briefly, genomic DNA was incubated at 37 °C with HaeIII, EcoRI, MseI, T4 ligase, ATP and a MseI Y adapter. The oligo nucleotide sequences containing barcodes are as follows: 5′-ACACTCTTTCCCTACACGACGCTCTTCCGATCTxxxx and 5′-TAAyyyyAGATCGGAAGAGCGTCGTGTAGGGAAAGAGTGT. The common adapters are as follows: 5′-TAAAGATCGGAAGAGCGGTTCAGCAGGAATGCCGAG and 5′-CTCGGCATTCCTGCTGAACCGCTCTTCCGATCT. After the restriction-ligation reactions, samples were further digested with the restriction enzymes HaeIII and EcoRI at 37 °C and then purified with an Agencourt AMPure XP (Beckman, Brea, CA, USA). PCR reactions were performed on the purified samples using a Phusion Master Mix universal primer and index primer. The polymerase chain reaction (PCR) products were purified again, and all PCR products were pooled to generate one library that included all 184 individuals. Fragments of 375–400 bp (with indexes and adapters) were isolated using a Gel Extraction Kit (Qiagen, Hilden, Germany). After being purified again, the fragments were diluted for sequencing and pair-end sequencing (PE, 150 bp) was performed using the Illumina HiSeq 4000 sequencing platform at Novogene Bioinformatics Technology Co. Ltd. (Beijing, China). After quality control, Illumina sequencing short reads were processed with Burrows-Wheeler Aligner’s maximal exact match (BWA-mem) [20], smatools-bcftools pipeline [21], vcftools [22] and several in-house scripts to call and filter SNPs and genotype all samples. 

### 2.5. Population Structural and Linkage Disequilibrium Analyses

The population structure was estimated by principal component analyses (PCA) using the software package GCTA [23] and linkage disequilibrium (LD) analysis was performed with PopLDdecay [24]. 

### 2.6. Genome-Wide Association Studies

We used GEMMA software [25] to perform genome-wide association studies for the ratio of TL and H using a generalized linear mixed model. The population genetic structure was considered to be a fixed effect and individual kinship was regarded as a random effect in the mixed linear model. The linear mixed model was constructed as follows:

y = X*α* + Z*β* +*ξ*+ *e* (1)

where y is the vector of the ratio of TL/H, X is the covariance matrix of the fixed effect, *α* is the estimated vector of the fixed effect, Z is the indicator matrix of the SNP, *β* is the effect of the SNP, *ξ* ~ *N*(0, *Kφ*^2^) is the polygenic effect, *K* is the genomic kinship matrix to adjust for kinship among samples, *φ*^2^ is the additive genetic variance and *e ~* (0, *δ_e_*^2^) is the random residual variance. The significance threshold was set to −log(1/n) [26].

### 2.7. RNA Extraction and Quantitative Real-Time PCR 

We selected 16 white sailfin mollies of about six months in age, including eight dwarf and eight normal sailfin mollies, at random from an ornamental fish market in Beijing. All fish samples were collected and raised in the same tank with fresh water at a temperature of 25 °C. After about two weeks the mollies were killed by rapidly cutting off the head, and the hepatopancreas and muscle were separated out immediately and stored at −80 °C for RNA extraction. Vertebrae from the end of the head to the anus were also cut off and dipped into trizol for about 25 min to remove the remaining muscle. After removing all muscle, the remaining vertebrae were stored at −80 °C for RNA extraction, or dipped into a new trizol solution for homogenation. After homogenation, the total RNA was extracted using the RNAprep Pure Tissue Kit (cat. #DP431, TIANGEN) according to the protocol supplied. The cDNA was obtained using a FastQuant RT Kit (cat. #KR106, TIANGEN) and quantitative real time PCR (qRT-PCR) was performed with 20 µL of the reaction system using SuperReal PreMix Plus (SYBR Green; cat. #FP205, TIANGEN) according to the protocols supplied. The thermal cycling procedure of qRT-PCR was as follows: 95 ℃ for 15 min followed by 40 cycles of 95 °C for 20 s, 57 °C for 20 s and 72 °C for 20 s, followed by a melting/dissociation curve stage for 6 s. Each sample had three technical duplications, and the expression levels of the target genes were adjusted by β-actin RNA expression. The primer sequences used in the present study are listed in Table S1. The 2^−∆∆Ct^ method was used to calculate the relative expression of each target gene [27]. Differences in expression were tested by *t*-tests using GraphPad Prism (Version 6; GraphPad Software, La Jolla, CA, USA). The significance thresholds were set at *p* < 0.05 and *p* < 0.01.

## 3. Results and Discussion

### 3.1. Phenotypic Data of the White Molly

After discarding a few ambiguous samples, 184 white mollies, including 92 normal white sailfin mollies (NWMs, 46 females and 46 males) and 92 dwarf white sailfin mollies (DWMs, 46 females and 46 males), were collected. The TL, H and TL/H were measured and calculated as shown in Table 1. From Table 1, we can see that the average TL/H of NWMs was 2.2256, which is significantly higher than that of DWMs (*p* < 0.01). 

### 3.2. Comparison of Skeletal Structures between the Dwarf Molly and the Normal Molly

**Figure 2:**
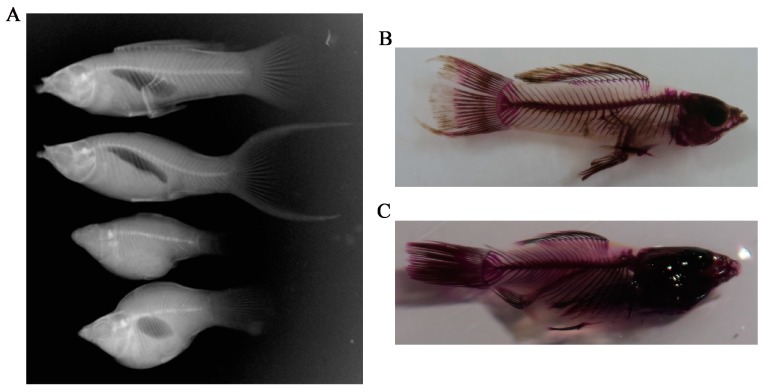
Skeletons of the normal molly and the dwarf molly. (**A**) Skeletons of normal and dwarf mollies under X-ray radioscopy; (**B**) skeleton of a normal molly using skeletal staining; (**C**) skeleton of a dwarf molly using skeletal staining.

In appearance, NWMs are slender and flat while DWMs have short bodies and convexly arched backs. The X-ray radioscopy inspection system and skeletal staining [18] were used to observe the difference in skeletal structure between the NWMs and DWMs. As shown in Figure 2, there were differences in the skeletal structures between the NWMs and DWMs, and the most obvious difference was in the spine. DWMs were short and their vertebrae were dysplastic in comparison with NWMs. The skeletal stainings were clear enough to count the vertebrae, as shown in Figure 2B,C. The white sailfin molly has 27–29 vertebrae and no significant difference (*p =* 0.16) was found in vertebrae number between the NWMs (n = 3) and DWMs (n = 5) in the present study. 

Dwarfism is a manifestation of abnormal skeletal development [2,28]. Skeletal dysplasia of tibia, cartilage and the spine can cause animal dwarfism [10,29,30]. Spinal deformities may play an important role in fish dwarfism [1]. Body deformity and dwarfism were often found to be associated with scoliosis, as demonstrated by Afonso et al. [31]. However, in the present study we did not find curved spines in the DWMs. Since no other reasonable factor was found to explain the dwarfism, we hypothesize that the dysplastic vertebrae seen in the DWMs are the main cause of dwarfism in the white sailfin molly, as has been suggested previously in a study of dwarfism in swordtail fish [1].

### 3.3. Sequencing Data Statistics and Quality Assessment

A summary of the next-generation sequencing is shown in Table S2. In total, 83.615 Gb of raw data and 83.612 Gb of clean data were obtained from 184 samples of white sailfin mollies, averaging 0.454 Gb per sample. For each sample, the GC distribution of the sequencing data was normal, and the value of Q30 was larger than 90.08% (Table S2). The results show that the sequencing quality was high and the data could be used for subsequent analysis. 

### 3.4. Single Nucleotide Polymorphism Filtering and Population Component Analysis

**Figure 3:**
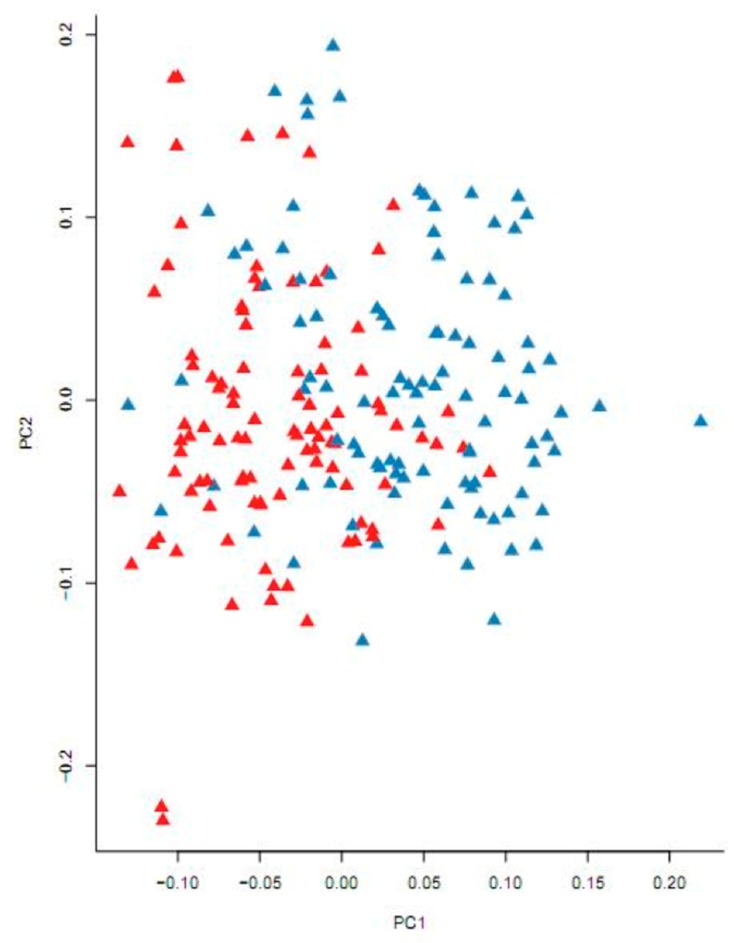
Principal component analysis performed with the GCTA package. Blue triangles represent NWMs and red triangles represent DWMs. Samples with the same body shape tend to come together, which implies that population stratification should be considered in the subsequent genome-wide association studies (GWAS). PC1: Principle component 1; PC2: Principle component 2.

In total, 3,021,852 SNPs were obtained from NWMs and DWMs using GBS technology. After removing the SNPs which fell outside of the following thresholds: sequencing depth below 4×, detection rate less than 20%, minimum allele frequency less than 0.05 and Hardy–Weiberg equilibrium (HWE) less than 0.00001, we obtained 113,814 high-quality SNPs for subsequent analysis with an SNP density of about 1.7 SNP per 10 kb in the whole genome. LD analysis was also performed with PopLDdecay [24] and the results are shown in Figure S1. With LD reduced to 50% of the highest original value (*r^2^* > 0.30) set as the threshold, the LD decay distance of the population is about 100 kb (Figure S1). Principle component analysis (PCA) is a useful method to reveal population stratification, which can cause false positives in GWAS [32]. In the present study, PCA was performed with GCTA [23] and the results are shown in Figure 3. From Figure 3, we can see significant population stratification, and so a univariate linear mixed model in GEMMA was performed to adjust for population stratification in the subsequent GWAS. 

### 3.5. Genome-Wide Association Studies

**Figure 4:**
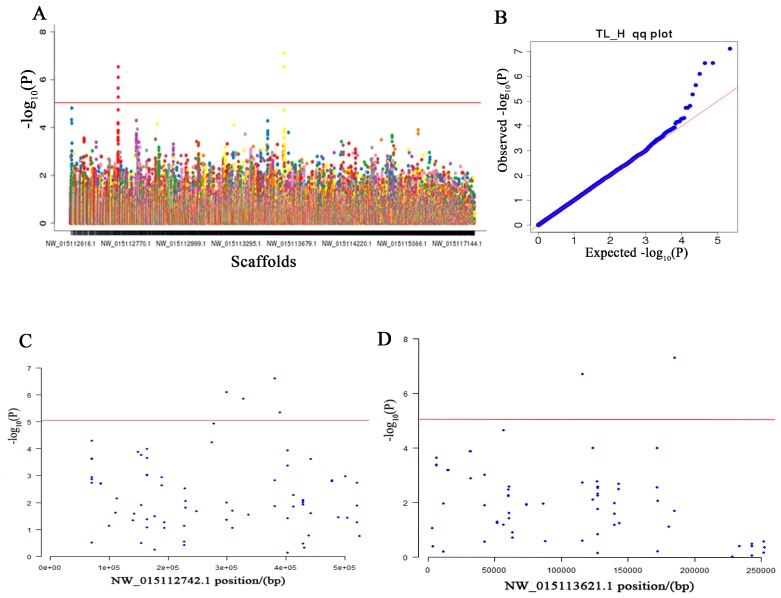
Manhattan plot (**A**) and quantile–quantile (Q–Q) plot (**B**) for the association of all single nucleotide polymorphisms (SNPs) with the ratio of tail fin length to height (TL/H). Regional plots (**C**,**D**) of the genome-wide significant SNPs. Six SNPs reached genome-wide significant level. In plots A, C and D the red line shows the *p*-value threshold of 8.79 × 10^−6^ (−log_10_(*p*-value) > 5.06).

In order to correct for the influence of individual size on phenotypic judgement, TL/H was calculated and used as the index of dwarfism in this study, and GEMMA [25] was used to test the association between the 113,814 SNPs and TL/H. Two regions on NW_015112742.1 and NW_015113621.1 were significantly associated with the trait (Figure 4, Table S3). The percent variance explained by the two loci was also calculated with GCTA [23] and the result is shown in Table S4. From Table S4, we can see that 99.3% of the phenotype variance can be explained by these two loci, which increases the credibility of our mapping results.

### 3.6. Prediction of Candidate Genes

To determine the candidate TL/H-related genes, significant SNPs were annotated with ANNOVAR [33] based on P_latipinna-1.0 (GCA_001443285.1; ftp://ftp.ncbi.nlm.nih.gov/genomes/all/GCF/001/443/285/GCF_001443285.1_P_latipinna-1.0/). Five genes, namely *ADAMTS like 1, NLRP 12 like, Larp 7, PPP3CA* and *Bank 1*, were annotated by the ranges from 20 kb upstream and downstream of the physical locations of those significant SNPs (Table S3).

**Figure 5:**
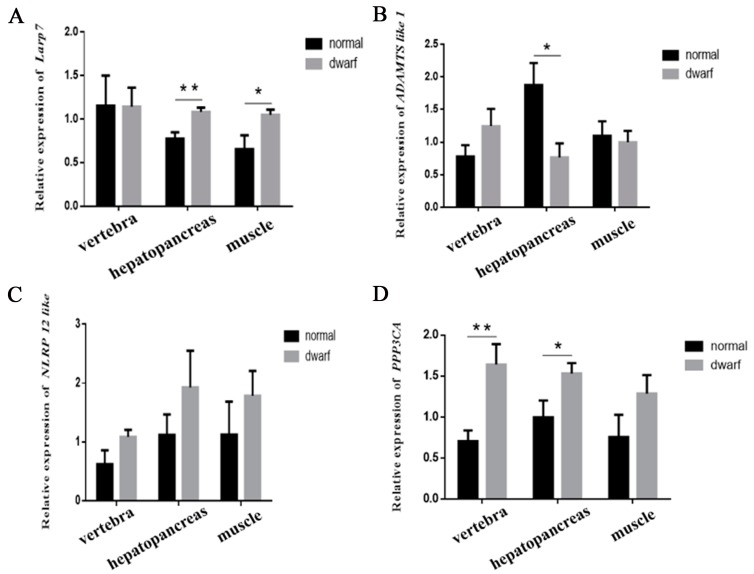
Comparison of the relative expression levels of genes in the candidate region in different tissues of the white normal sailfin molly (black bars) and the white dwarf sailfin molly (gray bars). (**A**) Relative expression level of *Larp7*; (**B**) relative expression level of *ADAMTS like 1*; (**C**) relative expression level of *NLRP 12-like*; (**D**) relative expression level of *PPP3CA*. Each bar represents the mean ± SE for each molly group (n = 8 for each group). * The values of gene expression levels are significantly different from each other (*p* < 0.05). ** The values of gene expression levels are significantly different from each other (*p* < 0.01).

Trait-causing genes often induce changes in gene expression levels, and so the gene expression levels of the five genes, except *Bank 1*, in the candidate region were determined by qRT-PCR (Figure 5). *Bank 1* (B-cell scaffold protein with ankyrin repeats 1) encodes a B-cell-specific scaffold protein, a novel substrate of tyrosine kinases, and its overexpression in B-cells can enhance calcium mobilization induced by B-cell antigen receptor (BCR) stimulation [34]. It has been reported that *Bank 1* is associated with systemic lupus erythematosus (SLE) [35,36]. The expression levels of the *Bank 1* gene were too low to be accurately determined in the three tissues examined in the present study, so no expression data of the *Bank 1* gene is shown here. There was no difference in the expression level of the *NLRP 12-like* gene between DWMs and NWMs in the vertebrae, hepatopancreas or muscle (Figure 5A). ADAMTS-like proteins may be enhancers of the ADAMTS proteases [37]. The *ADAMTS-like 1* gene, also known as *C9orf94*, *PUNCTIN*, *ADAMTSR1* or *ADAMTSL-1* in humans, encodes a novel ADAMTS-like molecule which lacks the typical domains of the ADAMTS family and is composed of only ADAMTS ancillary domains in the extracellular matrix [37]. A significant difference (*p* < 0.05) between the expression levels of *ADAMTS-like 1* in DWMs and NWMs was observed in the hepatopancreas, with the value observed for NWMs being greater than that observed for DWMs, but no difference was found in the vertebrae or muscle (Figure 5B). The *Larp7* gene encodes a member of the ribonucleoprotein domain family 7, which is a unit of the ribonucleoprotein complex 7SK snRNP. Some studies have suggested that *Larp7* mutations cause dwarf phenotypes [38,39,40]. Alamazi et al. [38] found that a seven-base homozygous repeat (c.1024_1030dupAAGGATA, p.T344K) on exon 8 of the *Larp7* gene, which causes a frameshift mutation and prematurely terminates translation, is an underlying mechanism of primordial dwarfism (PD), which is characterized by embryonic growth degeneration, facial deformity and mental impairment. Recently, two more mutations (c.1091_1094delCGGT and c.1045_1051dupAAGGATA) on the *Larp7* gene have been associated with PD [39]. In zebrafish, *Larp7* has also been found to play a crucial role in embryo development [40]. In the present study, the expression of the *Larp7* gene in DWMs was significantly higher (*p* < 0.05) than that in NWMs in the hepatopancreas and muscle, but not in the vertebrae (Figure 5C). *PPP3CA* encodes the alpha isoform of a subunit of calcineurin and is involved in neurodevelopmental diseases and systemic lupus erythematosus [35,36]. The expression of the *PPP3CA* gene in the hepatopancreas and vertebrae of DWMs was significantly (*p* < 0.05) or extremely significantly (*p* < 0.01) higher than that in NWMs (Figure 5D). The data also showed that DWMs possessed a higher expression level of the *PPP3CA* gene in muscle compared with NWMs, despite the fact that the difference was not significant (Figure 5D; *p* = 0.16). 

Some factors, such as fish developmental stage and potential differences in food intake between the two phenotypes, may affect gene expression levels. Although there is no significant difference between the two phenotypes of mollies in the expression of the *NLRP 12-like* gene at an age of six months, there may be differences at other stages, so we cannot exclude the role of *NLRP 12-like* in phenotypic formation based solely on this study. In comparison with NWMs, dwarf individuals may not be able to easily get food due to their deformed mouths and inflexible bodies, which can have impacts on gene expression. In order to reduce this effect, for the two-week duration of this study we fed all fish two times a day and evenly distributed enough food in the pool to ensure that all individuals could get enough food. 

## 4. Conclusions

We found that the white sailfin molly has 27–29 vertebrae in our study. The dwarf sailfin molly has a short and dysplastic spine in comparison with the normal sailfin molly, which indicated that the arrested longitudinal development of the vertebrae could be the main cause of the dwarf trait. Six SNPs significantly associated with pygmy traits were screened in the white sailfin molly, located at NW_015112742.1 and NW_015113621.1. According to the annotation of gene function and relative expression levels, we suggest *PPP3CA* and *Larp7* as the most important candidate dwarf-causing genes to be further investigated. 

## Figures and Tables

**Table 1:** Phenotype differences between normal and dwarf white sailfin mollies.

Character	NWM	DWM
Torso Lenght (cm)	3.6137 ± 0.0451 **	2.1828 ± 0.0451
Height (cm)	1.6264 ± 0.0211 *	1.5666 ± 0.0211
TL/H	2.2256 ± 0.0244 **	1.4097 ± 0.0244

Note: NWM is the abbreviation of normal white sailfin molly and DWM means dwarf white sailfin molly. * Indicates a significant difference (*p* < 0.05) between NWMs and DWMs; ** Indicates an extremely significant difference (*p* < 0.01) between NWMs and DWMs.

## Supplementary Materials

The following are available online at https://www.mdpi.com/2073-4425/10/6/418/s1, Table S1: The primer sequences for quantitative real-time PCR. Table S2: Summary of high-throughput sequencing data and quality control. Table S3: Significant SNPs associated with TL/H and candidate genes. Table S4: Estimated variance explained by the significant SNPs. Figure S1: Linkage disequilibrium (LD) decay distance of the population.
