# Supplementary material for: Identification of Loci Controlling the Dwarfism Trait in the White Sailfin Molly (Poecilia latipinna) Using Genome-Wide Association Studies Based on Genotyping-By-Sequencing
Source: Genes (Basel). 2019 May 30;10(6):418. doi: 10.3390/genes10060418 (PMC6628085; doi:10.3390/genes10060418)
Supplement: Supplementary file 1 [file genes-10-00418-s001.pdf]

Supplementary Materials

Table S1. The primers sequences for quantitative real-time PCR

Gene names	Primer sequences (5'-3')	Product length (bp)	*T_a (°C)
Larp7	#F: TCGGACGGTCTATGTGGAACTTT	117	59
	R: CCGGTACTCCTGTATCTGGGTAT		
NLRP12 like	F: CTCTTTGCACTGCGATTCGG	101	58
	R: CGTCAAATCAGCGAGTGACA		
ADAMTS like1	F: ACTTCTTCCCTTGCTCCGTCA	163	59
	R: CAAGGCTCCATGTTGCACTCC		
PPP3CA	F: ACATCCACGGGCAGTTCTTT	180	57
	R: TCCCACGCAGCAAAAACAGT		
β-actin	F: TCGTGCTGTCTTCCCATCCATCG	155	58
	R: AGTTGGTCACAATACCGTGCT		

#F is forward primer and R is reverse primer; *T_a means optimal annealing temperature (°C).

Table S2. Summary of high-throughput sequencing data and quality control

Sample ID	Raw Base (bp)	Clean Base (bp)	Effective Rate (%)	Error Rate (%)	Q20 (%)	Q30 (%)	GC Content (%)
1	317,220,192	317,212,704	100	0.01	97.66	94.47	39.9
3	321,016,896	321,008,832	100	0.01	97.75	94.68	40.34
4	278,633,376	278,623,008	100	0.01	97.69	94.53	40.41
5	324,595,584	324,584,928	100	0.01	97.81	94.81	40.03
6	367,253,856	367,238,592	100	0.01	97.89	95	40.11

7	256,450,464	256,444,704	100	0.01	97.8	94.73	40.55
8	270,386,496	270,379,584	100	0.02	97.46	93.9	40.59
9	277,721,856	277,709,472	100	0.01	97.84	94.94	40.37
10	286,418,016	286,410,240	100	0.01	97.7	94.58	40.31
11	291,032,352	291,022,272	100	0.01	97.81	94.83	40.48
13	392,335,488	392,325,696	100	0.01	97.82	94.83	40.51
14	391,539,744	391,532,256	100	0.01	97.75	94.59	39.91
15	324,397,440	324,386,496	100	0.01	97.61	94.33	40.13
17	295,349,184	295,341,408	100	0.01	97.64	94.31	40.45
18	302,860,224	302,851,296	100	0.02	97.56	94.18	40.54
19	353,878,848	353,868,480	100	0.01	97.73	94.59	40.63
20	330,162,336	330,146,784	100	0.01	97.78	94.81	40.32
21	295,197,408	295,189,056	100	0.01	97.78	94.75	40.14
22	281,741,184	281,730,528	100	0.01	97.7	94.6	39.94
23	334,146,816	334,130,400	100	0.01	97.72	94.71	40.17
24	275,288,256	275,279,616	100	0.01	97.8	94.81	40.41
25	285,104,160	285,095,232	100	0.02	97.41	93.77	40.3
26	298,726,272	298,716,480	100	0.01	97.66	94.39	40.51
28	374,263,776	374,248,800	100	0.01	97.85	94.88	40.45
29	284,871,168	284,863,680	100	0.02	97.56	94.14	40.25
30	321,310,368	321,296,256	100	0.01	97.82	94.91	40.42
31	317,884,032	317,870,784	100	0.01	97.82	94.9	40.38
32	278,076,384	278,069,760	100	0.01	97.82	94.8	40.53
33	309,106,656	309,100,032	100	0.01	97.78	94.78	40.05
34	261,889,632	261,881,280	100	0.02	97.53	94.09	40.38
35	258,428,448	258,421,824	100	0.02	97.34	93.64	40.19
36	292,529,376	292,520,736	100	0.01	97.77	94.69	40.2

37	271,480,320	271,474,560	100	0.02	97.62	94.32	40.12
38	227,837,664	227,833,056	100	0.02	96.79	92.3	40.37
39	373,445,856	373,431,168	100	0.01	97.78	94.74	40.31
40	333,761,760	333,749,664	100	0.01	97.79	94.75	40.45
41	350,207,136	350,200,800	100	0.01	97.75	94.59	39.76
42	401,390,784	401,385,888	100	0.01	97.85	94.81	40.25
43	343,572,192	343,567,584	100	0.01	97.76	94.58	40.34
44	449,061,408	449,052,768	100	0.01	97.91	94.95	40.02
45	396,784,512	396,777,600	100	0.01	97.94	95.05	40.02
46	314,345,664	314,340,480	100	0.01	97.86	94.78	40.33
47	335,485,728	335,481,120	100	0.02	97.53	93.95	40.34
48	310,036,608	310,030,848	100	0.01	97.9	95	40.2
49	317,831,328	317,826,144	100	0.01	97.76	94.64	40.14
50	318,410,208	318,403,296	100	0.01	97.87	94.88	40.14
51	420,452,928	420,444,576	100	0.01	97.92	94.98	40.34
52	418,849,920	418,845,024	100	0.01	97.82	94.66	39.79
53	374,141,088	374,135,904	100	0.01	97.72	94.47	40.19
54	347,537,088	347,533,920	100	0.01	97.72	94.39	40.36
55	369,340,128	369,334,368	100	0.01	97.64	94.28	40.38
56	385,918,272	385,911,936	100	0.01	97.81	94.7	40.26
57	415,791,936	415,781,856	100	0.01	97.86	94.93	40.12
58	330,922,080	330,916,032	100	0.01	97.86	94.86	39.94
59	361,462,464	361,455,840	100	0.01	97.8	94.72	39.84
60	387,781,056	387,775,584	100	0.01	97.8	94.81	40.03
63	347,288,256	347,279,904	100	0.01	97.88	94.92	40.2
66	334,733,472	334,730,880	100	0.02	97.48	93.83	40.27
67	385,789,536	385,783,200	100	0.01	97.75	94.5	40.44

68	434,794,464	434,786,112	100	0.01	97.94	94.99	40.25
69	383,739,552	383,734,080	100	0.02	97.64	94.22	40.23
70	336,230,496	336,223,584	100	0.01	97.9	95	40.1
71	387,734,976	387,726,624	100	0.01	97.89	94.98	40.25
72	354,745,152	354,738,528	100	0.01	97.85	94.81	40.38
73	367,083,648	367,077,312	100	0.01	97.86	94.87	39.97
75	343,641,024	343,635,552	100	0.01	97.63	94.22	40.22
77	338,492,736	338,490,720	100	0.02	97.39	93.66	40.03
78	345,290,688	345,286,080	100	0.01	97.82	94.73	40.02
79	336,875,328	336,871,584	100	0.01	97.69	94.4	40.12
80	250,888,608	250,886,016	100	0.02	96.83	92.28	40.22
81	405,188,928	405,179,424	100	0.01	97.85	94.83	40.2
82	380,061,792	380,056,896	100	0.01	97.82	94.77	40.16
83	548,269,056	548,208,288	99.99	0.02	97.05	92.89	39.7
84	532,686,240	532,625,184	99.99	0.02	97.25	93.31	40.39
85	543,561,696	543,499,776	99.99	0.02	97.09	92.93	40.58
86	554,194,656	554,135,328	99.99	0.02	97.25	93.33	40.16
87	521,116,128	521,055,648	99.99	0.01	97.34	93.55	40.05
88	392,190,336	392,184,864	100	0.01	97.66	94.49	39.8
89	321,564,960	321,559,776	100	0.01	97.7	94.62	40.14
90	376,139,520	376,133,472	100	0.01	97.66	94.48	40.43
91	424,201,248	424,193,184	100	0.01	97.82	94.84	40.11
93	374,817,024	374,807,232	100	0.01	97.84	94.91	40.1
94	332,396,064	332,390,880	100	0.01	97.8	94.76	40.52
96	369,003,744	368,996,544	100	0.02	97.41	93.84	40.36
97	415,198,080	415,189,440	100	0.01	97.83	94.92	40.41
98	477,324,576	477,272,160	99.99	0.02	97.28	93.33	40.91

99	384,494,976	384,487,488	100	0.01	97.73	94.66	40.41
100	233,142,912	233,138,592	100	0.01	97.82	94.84	40.5
101	392,210,784	392,201,280	100	0.01	97.84	94.88	40.61
103	390,262,176	390,256,992	100	0.01	97.75	94.61	39.91
104	394,049,952	394,045,056	100	0.01	97.61	94.36	40.2
105	414,101,376	414,094,464	100	0.01	97.68	94.43	40.45
106	400,676,256	400,670,496	100	0.01	97.56	94.2	40.57
107	400,753,440	400,746,816	100	0.01	97.73	94.63	40.47
108	493,329,888	493,316,064	100	0.01	97.82	94.91	40.38
110	352,030,464	352,026,432	100	0.01	97.78	94.77	40.06
111	363,957,696	363,949,920	100	0.01	97.69	94.6	40.03
112	371,017,440	371,009,088	100	0.01	97.69	94.69	40.1
113	518,162,688	518,105,088	99.99	0.02	96.76	92.12	40.69
114	515,427,264	515,376,288	99.99	0.01	97.31	93.56	40.52
115	513,490,464	513,433,440	99.99	0.02	97.11	93.05	40.53
116	520,632,864	520,577,856	99.99	0.01	97.26	93.36	40.66
117	562,661,280	562,589,568	99.99	0.01	97.23	93.3	40.75
118	525,842,496	525,777,696	99.99	0.02	97.15	92.99	40.27
119	651,476,736	651,406,464	99.99	0.02	97.03	92.74	40.14
120	522,013,248	521,964,288	99.99	0.02	97.01	92.64	40.49
121	565,964,640	565,907,328	99.99	0.02	96.92	92.54	40.56
122	600,602,400	600,535,008	99.99	0.02	97.19	93.14	40.62
123	610,720,704	610,653,888	99.99	0.01	97.25	93.46	40.49
124	603,159,264	603,086,400	99.99	0.02	97.22	93.29	40.39
125	582,092,928	582,024,672	99.99	0.02	97.13	93.12	40.27
126	540,431,712	540,369,504	99.99	0.01	97.17	93.34	40.5
127	566,260,128	566,195,328	99.99	0.01	97.27	93.43	40.47

128	546,285,888	546,224,256	99.99	0.02	96.73	92.04	40.5
129	581,735,808	581,671,584	99.99	0.02	97.03	92.76	40.65
130	615,193,632	615,123,936	99.99	0.02	97.31	93.45	40.56
131	710,278,848	710,208,288	99.99	0.02	96.97	92.58	40.41
132	617,836,032	617,761,728	99.99	0.01	97.26	93.5	40.57
134	604,994,400	604,916,640	99.99	0.01	97.3	93.54	40.49
135	630,000,000	629,936,640	99.99	0.02	97.23	93.28	40.67
136	565,358,976	565,295,616	99.99	0.02	97.22	93.32	40.23
137	579,570,336	579,507,552	99.99	0.02	96.92	92.48	40.34
138	541,691,136	541,633,248	99.99	0.02	96.61	91.82	40.28
139	534,790,656	534,731,616	99.99	0.02	97.18	93.16	40.25
140	528,407,424	528,348,960	99.99	0.02	97	92.69	40.18
141	451,296,576	451,262,304	99.99	0.03	95.85	90.08	40.37
142	553,754,880	553,693,536	99.99	0.02	97.19	93.23	40.34
143	572,937,408	572,882,976	99.99	0.02	97.18	93.19	40.4
144	801,085,536	801,054,144	100	0.01	97.65	94.66	39.66
145	610,981,344	610,956,288	100	0.01	97.75	94.87	40.27
146	573,090,624	573,069,312	100	0.01	97.65	94.65	40.44
147	704,802,240	704,774,880	100	0.01	97.78	94.97	40.2
148	584,164,224	584,142,912	100	0.01	97.82	95.07	39.96
149	543,055,392	543,032,064	100	0.01	97.78	94.91	40.85
150	568,375,488	568,356,480	100	0.01	97.41	94.06	40.88
151	657,779,616	657,750,528	100	0.01	97.82	95.1	40.55
152	621,419,616	621,395,712	100	0.01	97.75	94.88	40.2
197	668,331,360	668,307,744	100	0.01	97.79	94.98	40.53
199	594,744,192	594,723,744	100	0.01	97.76	94.93	40.48
200	693,260,064	693,231,552	100	0.01	97.71	94.75	39.92

208	603,519,264	603,494,784	100	0.01	97.47	94.24	40.14
212	524,585,952	524,570,400	100	0.01	97.59	94.45	40.52
302	507,594,528	507,578,976	100	0.01	97.47	94.23	40.71
310	595,834,848	595,808,640	100	0.01	97.71	94.78	40.62
311	582,527,232	582,507,072	100	0.01	97.75	94.96	40.49
312	590,889,888	590,872,608	100	0.01	97.75	94.9	40.41
313	601,373,376	601,349,184	100	0.01	97.68	94.76	40.36
314	579,746,592	579,727,008	100	0.01	97.72	94.91	40.58
315	572,073,984	572,051,520	100	0.01	97.78	94.99	40.53
316	541,588,608	541,576,224	100	0.01	97.38	93.94	40.67
317	478,838,304	478,822,464	100	0.01	97.64	94.56	40.91
318	593,068,032	593,044,992	100	0.01	97.8	94.98	40.59
319	585,704,160	585,681,696	100	0.02	97.54	94.31	40.38
320	556,405,056	556,388,064	100	0.01	97.75	94.97	40.52
321	548,791,200	548,771,040	100	0.01	97.79	95.05	40.48
322	530,932,320	530,910,432	100	0.01	97.77	94.89	40.84
323	577,502,784	577,476,576	100	0.01	97.69	94.79	40.27
324	496,938,240	496,918,656	100	0.01	97.52	94.3	40.39
331	559,558,656	559,540,224	100	0.02	97.31	93.81	40.3
332	593,042,112	593,021,952	100	0.01	97.75	94.88	40.28
333	604,753,344	604,731,456	100	0.01	97.59	94.47	40.35
334	491,601,024	491,583,744	100	0.02	96.82	92.6	40.59
335	585,653,184	585,627,552	100	0.01	97.69	94.78	40.38
336	514,763,712	514,746,144	100	0.01	97.71	94.83	40.36
337	590,194,944	590,190,912	100	0.02	96.86	92.55	40.11
338	445,226,688	445,224,384	100	0.02	96.88	92.54	40.45
339	438,502,752	438,500,736	100	0.02	96.79	92.4	40.52

340	444,259,872	444,256,416	100	0.02	97.02	92.96	40.49
341	633,165,696	633,157,056	100	0.01	97.1	93.3	40.41
342	522,342,432	522,337,536	100	0.02	97.05	93.09	40.23
343	558,872,064	558,866,592	100	0.02	96.98	92.94	40.18
345	494,824,032	494,817,696	100	0.01	97	93.13	40.43
347	578,546,784	578,540,736	100	0.01	97.1	93.22	40.48
353	572,908,032	572,905,152	100	0.02	96.63	91.98	40.53
354	620,124,192	620,117,856	100	0.02	96.91	92.65	40.78
356	574,754,112	574,747,200	100	0.02	97.12	93.22	40.59
358	485,326,944	485,325,216	100	0.02	96.84	92.47	40.47
359	543,460,608	543,451,968	100	0.01	97.06	93.23	40.53
C4	526,326,624	526,319,424	100	0.01	97.11	93.28	40.44
Q13	491,250,240	491,244,768	100	0.02	97.04	93.05	40.61
Q2	544,711,392	544,705,920	100	0.02	97.05	93.11	40.13
Q3	516,009,600	516,007,008	100	0.02	96.81	92.41	40.39
Q4	501,535,296	501,533,568	100	0.02	96.52	91.75	40.27
Q5	550,676,736	550,670,976	100	0.02	97	92.95	40.17
Summary	83,615,399,712 (0.454Gb/per)	83,611,591,776 (0.454Gb/per)	>99.99	>0.01	>95.85	>90.08	39.66 ~ 40.91

Table S3. Significant SNPs associated with TL/H and candidate genes

No.	Scaffold.	Position (bp)	SNP (ref/alt)	P-value	$-\log_{10}$(P_value)	Annotation	Position (bp)	The beginning of CDS (bp)

1	NW_015113621.1	115528	C/T	2.872274e-07	6.54	*PPP3CA*	50056..155150	154887
2		184881	C/T	7.633129e-08	7.12	Bank 1	169580..191914	169580
3	NW_015112742.1	299448	C/T	7.880186e-07	6.10	*ADAMTS like 1*	252489..366156	253189
4		327389	A/G	2.244672e-06	5.65	*NLRP 12 like*	391279..397395	391502
5		381425	A/G	2.905988e-07	6.54	*Larp7*	398368..404485	398898
6		389538	G/A	5.293656e-06	5.28			

Table S4. Estimate variance explained by the significant SNPs

Source	Variance	SE
V(G)	382.158502	0
V(e)	2.634312	0.000001
Vp	384.792814	0.000001
V(G)/Vp	0.993154	0
logL	-853002.565	
logL0	9.132	
LRT	0	
df	1	
Pval	0.5	
n	184	

*Note: The results are obtained by GCTA-GREML (gcta_1.92.1beta6).

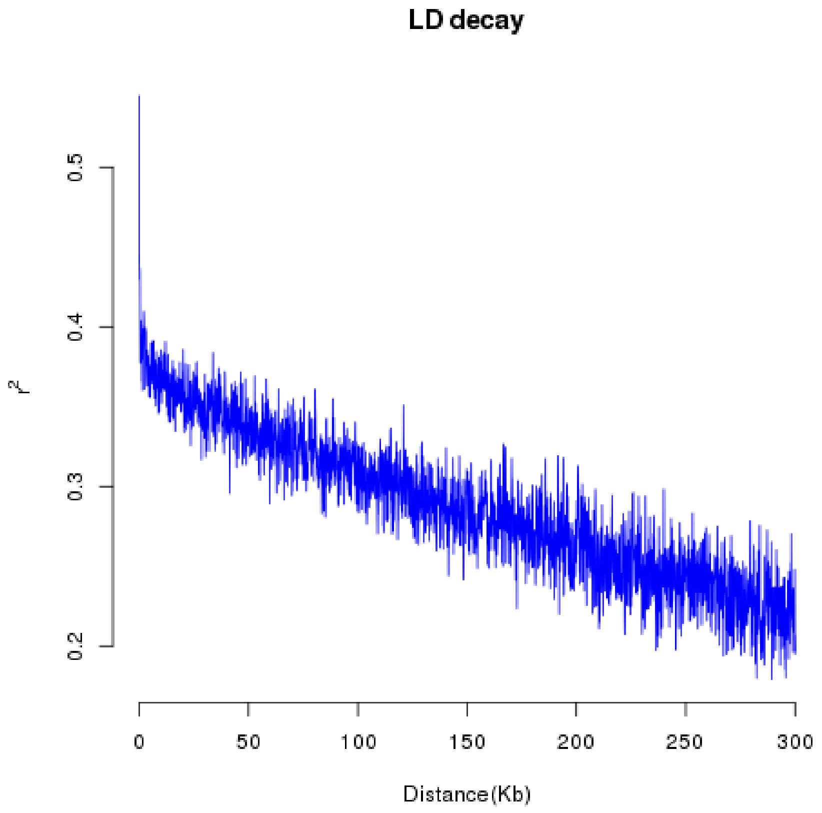

Figure S1: Linkage disequilibrium (LD) decay distance of the population.

The LD analysis was also performed with PopLDdecay [24]. With LD reduced to 50% of the highest original value ($r^2 > 0.30$) as its threshold, the LD decay distance of the population is about 100 kb.
